# Milk-Derived Small Extracellular Vesicles Promote Osteogenic Differentiation and Inhibit Inflammation via microRNA-21

**DOI:** 10.3390/ijms241813873

**Published:** 2023-09-08

**Authors:** Runyuan Liu, Shuo Liu, Saixuan Wu, Meng Xia, Wanqing Liu, Lina Wang, Ming Dong, Weidong Niu

**Affiliations:** School of Stomatology, Dalian Medical University, Dalian 116044, China

**Keywords:** chronic apical periodontitis, *miR-21*, milk, small extracellular vesicles

## Abstract

Chronic apical periodontitis (CAP) is a disease with characteristics of inflammation and bone loss. In this study, our objective was to examine the function of small extracellular vesicles (sEVs) obtained from milk in encouraging osteogenic differentiation and inhibiting inflammation by *miR-21* in CAP. The expression of *miR-21* was detected using qRT-PCR in human CAP samples. The impact of *miR-21* on the process of osteogenic differentiation was investigated using CCK-8, qRT-PCR, immunofluorescence staining, and Western blot analysis. The evaluation of RAW 264.7 cell polarization and the assessment of inflammatory factor expression were conducted through qRT-PCR. The influence of sEVs on MC3T3-E1 cells and RAW 264.7 cells was examined, with a particular emphasis on the involvement of *miR-21*. In human CAP samples, a decrease in *miR-21* expression was observed. *MiR-21* increased the expression of osteogenesis-related genes and M2 polarization genes while decreasing the expression of M1 polarization genes and inflammatory cytokines. Treatment with milk-derived sEVs also promoted osteogenesis and M2 polarization while inhibiting M1 polarization and inflammation. Conversely, the addition of *miR-21* inhibitors resulted in opposite effects. Our results indicated that sEVs derived from milk had a positive effect on bone formation and activation of anti-inflammatory (M2) macrophages and simultaneously reduced inflammation by regulating *miR-21* in CAP.

## 1. Introduction

Chronic apical periodontitis (CAP) is a disease with characteristics of long-term inflammation and alveolar bone loss [1]. The prevalence of CAP varies due to factors such as region, socioeconomic status of the country, and research methods. The results of a meta-analysis indicate that the prevalence of CAP is around 52% [2]. When teeth are subjected to trauma or caries, various microorganisms may enter the pulp cavity or root canal system. These microorganisms release bacterial lipopolysaccharides (LPS) and inflammatory cytokines, leading to inflammation and even necrosis of the dental pulp tissue, ultimately causing CAP [3]. When root canal treatment is incomplete, it can even lead to loosening or loss of teeth, reducing the quality of life of patients [2]. CAP is considered a potential risk for systemic diseases, including cardiovascular diseases [4], obstructive sleep apnea [5,6], and depression [7]. Although CAP has been widely studied, there is limited research on the repair of bone defects associated with CAP [1,8]. Therefore, it is crucial to find novel therapeutic agents that can promote alveolar bone formation and inhibit inflammation in patients with CAP.

*MicroRNA-21 (miR-21)* is a specific *microRNA* that plays a vital role in osteogenesis [9] and inflammation [10]. Knockout of the *miR-21* gene in mice diminished their bone repair capacity, whereas the addition of *miR-21* agonists improved bone defect repair [11]. Other studies have shown that *miR-21* promotes the expression of osteogenic markers such as *Runx2, OPN*, and *OCN*, thereby facilitating bone formation [12]. Furthermore, *miR-21* inhibits the expression of inflammatory cytokines *IL-6, IL-1β*, and *IL-8* while increasing the expression of anti-inflammatory cytokine *IL-10* through the *PDCD4/NF-κB* signaling pathway [13]. The absence of *miR-21* leads to more severe gingival inflammation and alveolar bone resorption, indicating that it may be an intervention target for controlling periodontitis [14]. Both periodontitis and CAP have features of inflammation and bone destruction [15]. However, no studies have reported on the role of *miR-21* in CAP.

Small extracellular vesicles (sEVs) are lipid bilayer vesicles with an average diameter smaller than 200 nm [16]. Compared to cell-based therapies, sEVs display low immunogenicity, optimal biocompatibility, and analogous biological properties to their originating cells [17,18]. These avoid the limitations of conventional cell therapy, including immune rejection, long-term safety risks, and cellular senescence [19]. They can be isolated from various cells and body fluids such as milk, blood, and urine [20]. Common methods for sEV isolation include differential ultracentrifugation, size exclusion chromatography, and ultrafiltration [21]. sEVs contain many genetic materials, such as *DNA*, *mRNA*, *microRNA*, and protein [22]. Bone mesenchymal stem cell (BMSC)-derived sEVs transfected with *miR-23a-3p* mimics promote M2 macrophage polarization and facilitate bone healing while reducing inflammation through activation of the *IRF1* and *NF-κB* pathways [23]. Similarly, adipose mesenchymal stem cell (ASC)-derived sEVs enriched with *miR-451a* exhibit significant efficacy in promoting bone healing, shifting macrophages from the M1 to M2 phenotype and suppressing inflammation via the *miR-451a/MIF* signaling pathway [24]. Milk-derived sEV-mediated delivery of *miR-31-5p* improves endothelial cell functions, accelerates angiogenesis, and enhances the healing of diabetic wounds [25]. Compared to sEVs derived from cellular and other bodily fluid sources, milk-derived sEVs can be efficiently mass-produced utilizing abundant milk resources [26]. These vesicles come with reduced production expenses, improved intestinal permeability, and heightened safety profiles and can be administered continuously without provoking allergic reactions [27]. Meanwhile, studies have revealed that sEVs derived from cow’s milk contain abundant *miR-21* [28]. These findings offer a promising therapeutic strategy for bone repair in an inflammatory environment. Thus, it is worth investigating whether sEVs derived from bovine milk can exert their effects on CAP through *miR-21*.

In this study, our objective was to examine the role of milk-derived sEVs in CAP, with the hypothesis that they promoted osteogenic differentiation and inhibited inflammation by modulating *miR-21*. We discovered that *miR-21* increased the expression of osteogenesis-related genes and M2 polarization genes while decreasing the expression of M1 polarization genes and inflammatory cytokines. To further investigate whether milk-derived sEVs exert their effects through *miR-21*, we successfully isolated sEVs from *cow’s* milk and analyzed the effect of sEVs derived from milk on promoting osteogenic differentiation of MC3T3-E1 cells, inducing M2 polarization of RAW 264.7 cells, and suppressing inflammation via *miR-21*. Our results indicated that *miR-21* might be a potential therapeutic agent for sEVs in the treatment of CAP.

## 2. Results

### 2.1. Expression of miR-21 in CAP

**Figure 1:**
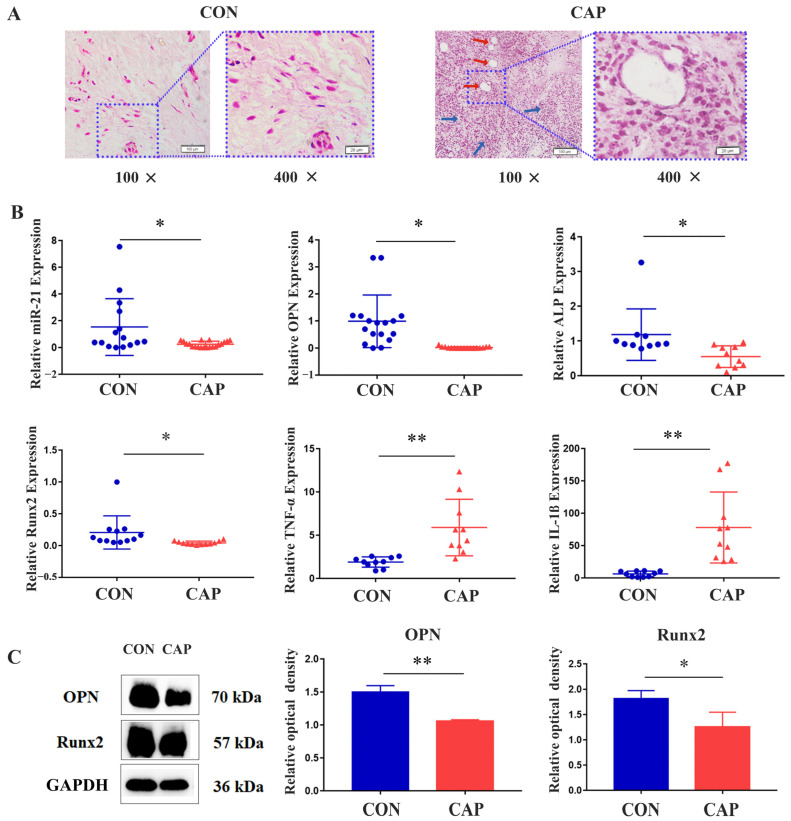
Identification of clinical samples and expression of *miR-21* in *human* CAP. (**A**) Histological analysis using HE staining confirmed the extraction of clinical samples: blood vessels (red arrows); inflammatory cells (blue arrows); scale bars, 100 μm, 20 μm. (**B**) The qRT-PCR method was used to detect the expression of *miR-21*, osteogenic differentiation marker genes (*OPN*, *ALP*, and R*unx2*), and inflammatory factors *TNF-α* and *IL-1β*. (**C**) Western blotting was used to detect the expression of osteogenic differentiation marker proteins. * *p* < 0.05, ** *p* < 0.01.

From the clinical samples, we selected 25 cases of CAP samples and 25 cases of healthy periapical membrane tissues. These cases, aged 24 to 55, included 23 females and 27 males, showing no significant gender or age differences (*p* > 0.05). CAP group lesions were taken from patients with uncontrolled apical periodontitis, undergoing periradicular curettage or tooth extraction, with harvested lesions processed after patient consent. Normal apical tissue in the CON group came from patients needing third molar or healthy premolar extraction for orthodontic reasons. HE staining revealed that inflammatory cells and newly formed capillaries were present in the CAP group (Figure 1A). Analysis by qRT-PCR indicated a decrease in the expression of *miR-21* (*p*-value, 0.011; 95 percent confidence interval, 0.32 to 2.25) and the osteogenic markers *OPN*, *ALP*, and *Runx2* in the CAP samples compared to the CON group. Conversely, expression of the inflammatory cytokines *TNF-α* and *IL-1β* was found to be increased in the CAP group (Figure 1B). Western blot analysis indicated a significant decrease in the expression of OPN and Runx2 in the CAP group compared to the CON group (Figure 1C).

### 2.2. Expression of Osteogenic Factors and miR-21 during Differentiation of MC3T3-E1 Cells

**Figure 2:**
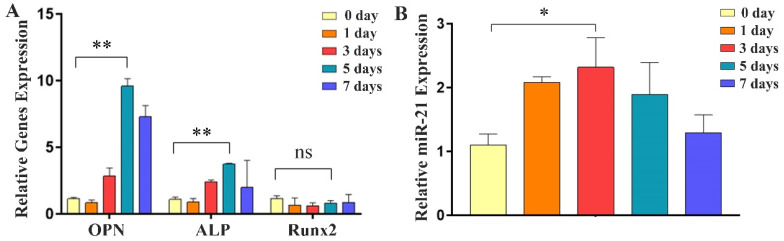
The expression of *miR-21* and osteogenic genes during the differentiation of MC3T3-E1 cells. (**A**) qRT-PCR was used to detect the expression of osteogenic differentiation factors at 0 to 7 d during the process of osteogenic differentiation. (**B**) The expression of *miR-21* was also examined using qRT-PCR during the same time period of osteogenic differentiation. * *p* < 0.05, ** *p* < 0.001, ns: not statistically significant.

During the osteogenic differentiation of MC3T3-E1 cells, qRT-PCR analysis was performed on days 0–7. The results revealed a gradual increase in the expression of osteogenic marker genes, *OPN* and *ALP*, with the highest expression observed on day 5 (Figure 2A). Furthermore, the expression of *miR-21* also exhibited a progressive increase during the osteogenic differentiation process and increased 2.3 times to reach a peak on day 3 (*p*-value, 0.013; 95 percent confidence interval, −2.01 to −0.42), occurring earlier than the peak expression of *OPN* and *ALP* (Figure 2B). These results demonstrated that *miR-21* was involved in osteogenic differentiation and might influence the expression of *OPN* and *ALP*.

### 2.3. Effects of miR-21 on the Proliferation and Osteogenic Differentiation of MC3T3-E1 Cells under LPS Stimulation

**Figure 3:**
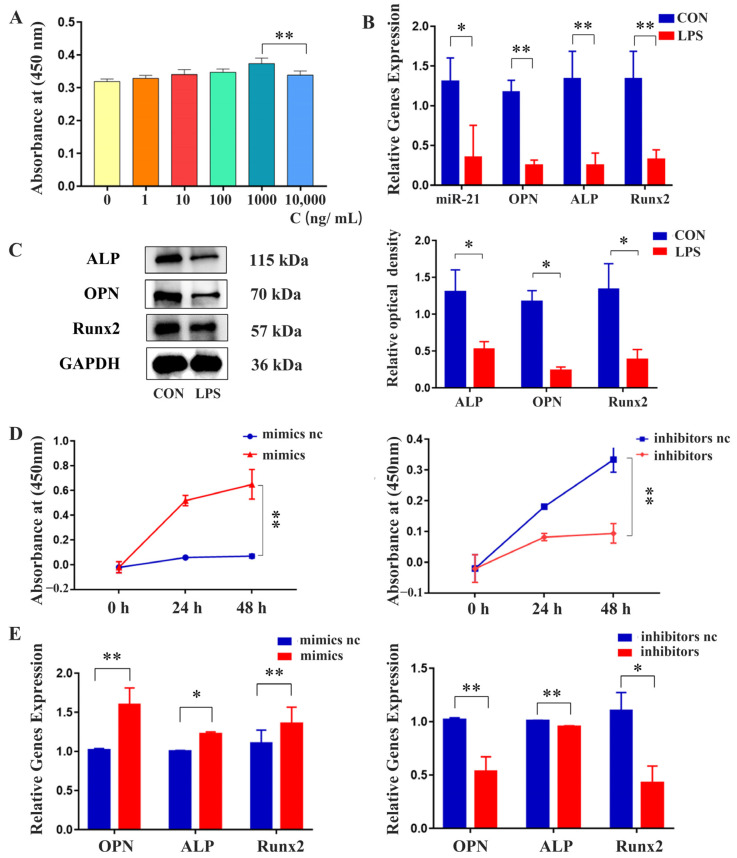
Effects of *miR-21* on the proliferation and differentiation of MC3T3-E1 cells under LPS stimulation. (**A**) The optimal concentration of LPS for induction of MC3T3-E1 cells was determined using the CCK-8 assay, 0 ng/mL (yellow), 1 ng/mL (orange), 10 ng/mL (red), 100 ng/mL (green), 1000 ng/mL (dark green), 10,000 ng/mL (blue). (**B**) The expression of osteogenic marker genes was assessed using qRT-PCR. (**C**) The expression of osteogenic proteins was detected using Western blot analysis. (**D**) The effect of *miR-21* on the proliferation of MC3T3-E1 cells was evaluated using CCK-8 assay. (**E**) The impact of *miR-21* on the expression of osteogenic differentiation genes was assessed using qRT-PCR. * *p* < 0.05, ** *p* < 0.01.

The results of the CCK-8 assay showed that a concentration of 1000 ng/mL LPS promoted the proliferation of MC3T3-E1 cells without inducing toxicity (Figure 3A). qRT-PCR analysis revealed that LPS-induced inflammatory conditions resulted in reduced gene expression levels of *OPN*, *ALP*, *Runx2*, and *miR-21* (Figure 3B). Western blot analysis confirmed that the protein expression levels of OPN, ALP, and Runx2 were reduced in the presence of LPS-mediated inflammation (Figure 3C). However, the CCK-8 assay revealed that MC3T3-E1 cells transfected with *miR-21* mimics exhibited increased proliferation compared to NC, which was decreased when MC3T3-E1 cells were transfected with *miR-21* inhibitors (Figure 3D). Additionally, qRT-PCR analysis indicated that the transfection of *miR-21* mimics increased the expression levels of *OPN*, *ALP*, and *Runx2.* Conversely, inhibiting *miR-21* led to a decrease in the expression of these osteogenic markers (Figure 3E).

### 2.4. miR-21 Promoted the Expression of Osteogenic Proteins in MC3T3-E1 Cells under LPS Stimulation

**Figure 4:**
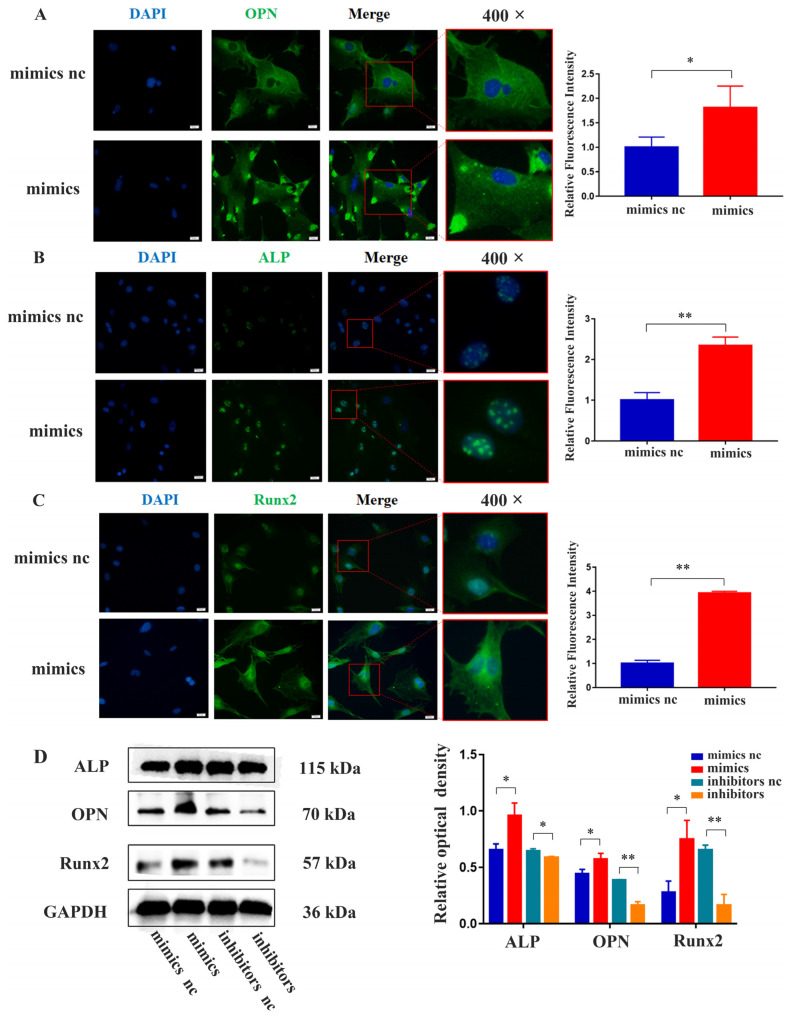
The effects of *miR-21* on the expression of osteogenic proteins in MC3T3-E1 cells under LPS stimulation. (**A**) Immunofluorescence staining was performed to observe the localization of OPN. (**B**) ALP. (**C**) Runx2; scale bar, 20 μm. (**D**) Western blot analysis examined the expression of osteogenic proteins in MC3T3-E1 cells following the transfection of *miR-21* under inflammatory conditions. * *p* < 0.05, ** *p* < 0.01.

The results of immunofluorescence staining demonstrated that the cytoplasmic fluorescence intensity of OPN was higher in the *miR-21* mimics group compared to the NC group. Additionally, the expression level of ALP in the nucleus was higher in the *miR-21* mimics group. Furthermore, the expression of Runx2 in both the nucleus and cytoplasm was higher in the *miR-21* mimics group (Figure 4A–C). Western blot analysis revealed that MC3T3-E1 cells transfected with *miR-21* mimics in the inflammatory environment exhibited increased protein levels of ALP, OPN, and Runx2, while inhibition of *miR-21* showed the opposite effect (Figure 4D).

### 2.5. miR-21 Regulated the Polarization and Expression of Inflammatory Factors in RAW 264.7 Cells under LPS Stimulation

**Figure 5:**
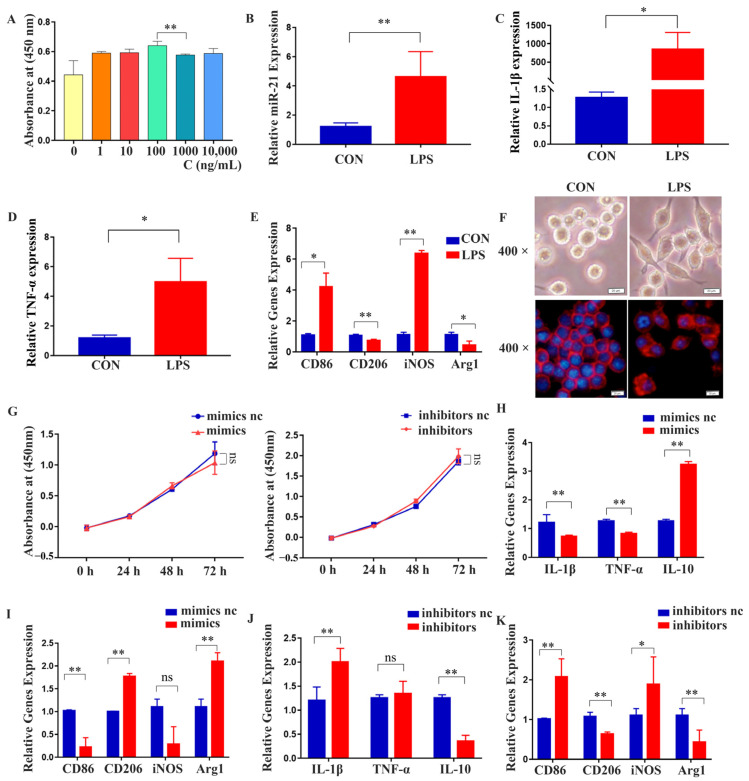
The effects of *miR-21* on RAW 264.7 cell polarization and expression of inflammatory factors under LPS stimulation. (**A**) CCK-8 assay was performed to determine the optimal concentration of LPS, 0 ng/mL (yellow), 1 ng/mL (orange), 10 ng/mL (red), 100 ng/mL (green), 1000 ng/mL (dark green), 10,000 ng/mL (blue). (**B**–**E**) qRT-PCR was conducted to assess the expression of *miR-21*, *IL-1β*, *TNF-α*, *CD86*, *iNOS*, *CD206*, and *Arg1* in an inflammatory environment. (**F**) The morphology of RAW 264.7 cells was observed under a light microscope and a fluorescence microscope; scale bar, 20 μm. (**G**) The effect of *miR-21* on the proliferation of RAW 264.7 cells was detected by CCK-8 assay. (**H**,**I**) After transfection with *miR-21* mimics, the expression levels of *IL-1β, TNF-α, CD86, iNOS, CD206*, and *Arg1* were analyzed in RAW 264.7 cells using qRT-PCR. (**J**,**K**) The opposite effect was observed when *miR-21* was inhibited. * *p* < 0.05, ** *p* < 0.01, ns: not statistically significant.

The results of the CCK-8 assay showed that a concentration of 1000 ng/mL LPS promoted the proliferation of RAW 264.7 cells without inducing toxicity (Figure 5A). qRT-PCR analysis revealed that under LPS stimulation, the expression of *miR-21*, inflammatory cytokines (*IL-1β* and *TNF-α*), and M1 polarization markers (*CD86* and *iNOS*) all increased (Figure 5B–E). RAW 264.7 cells exhibited irregular morphology and extended pseudopods in an inflammatory environment, as observed under a 400-times magnification optical microscope and a fluorescence microscope (Figure 5F). The transfection of *miR-21* mimics and inhibitors had minimal impact on RAW 264.7 cell proliferation (Figure 5G). However, transfection of *miR-21* mimics led to a decrease in the expression of inflammatory cytokines (*IL-1β* and *TNF-α*) and M1 polarization markers (*CD86* and *iNOS*), as revealed by qRT-PCR analysis. Additionally, there was an increase in the expression of M2 polarization markers (*CD206* and *Arg1*) and the anti-inflammatory cytokine *IL-10* (Figure 5H,I). Conversely, *miR-21* inhibition showed the opposite effect (Figure 5J,K).

### 2.6. Isolation and Identification of Milk-Derived sEVs and Their Effects on MC3T3-E1 Cells and RAW 264.7 Cells

**Figure 6:**
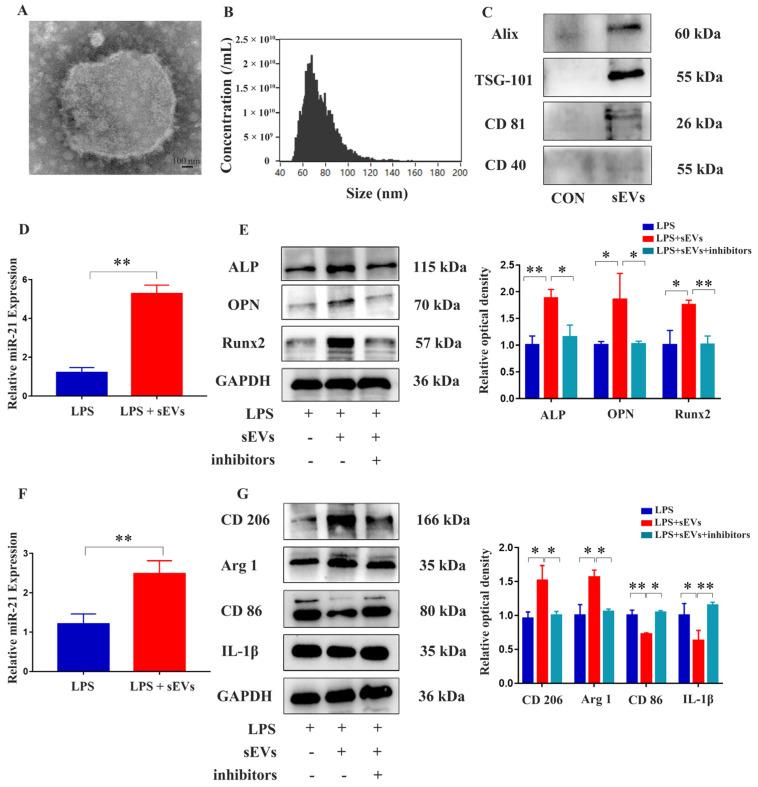
Identification of milk-derived sEVs and their effects on MC3T3-E1 cells and RAW 264.7 cells. (**A**) Observation of the morphology of sEVs under TEM; scale bar, 100 μm. (**B**) Nanoparticle flow cytometric analysis was performed to determine the size and concentration of sEVs. (**C**) Western blotting was used to analyze protein expression of sEV markers. (**D**) qRT-PCR analysis was performed to measure the expression of *miR-21* in MC3T3-E1 cells after the addition of sEVs. (**E**) Western blot analysis was conducted to assess the expression of ALP, OPN, and Runx2. (**F**) qRT-PCR analysis was performed to measure the expression of *miR-21* in RAW 264.7 cells after the addition of sEVs. (**G**) Western blot analysis examined the expression of IL-1β, CD86, and CD206. * *p* < 0.05, ** *p* < 0.01.

sEVs derived from *cow’s* milk were isolated using ultra-high-speed differential centrifugation. Morphological examination by transmission electron microscopy (TEM) showed that the sEVs were disc-shaped lipid bilayer structures (Figure 6A). Nano-flow cytometric analysis showed that the median particle size of sEVs was 72.2 nm, and the particle concentration was 2.01 × 10^10^/mL (Figure 6B). The positive markers CD81, TSG101, and Alix were highly expressed in the sEV group, while the negative marker CD40 was expressed at a low level (Figure 6C). These results indicated that we had extracted milk-derived sEVs successfully. In an inflammatory environment, it was found that expression of *miR-21* significantly increased when MC3T3-E1 cells were treated with milk-derived sEVs (Figure 6D). Western blot analysis revealed that sEVs promoted the expression of osteogenic differentiation markers, including ALP, OPN, and Runx2 in MC3T3-E1 cells, but this effect was blocked by inhibiting *miR-21* (Figure 6E,F). These results indicated that milk-derived sEVs promoted bone differentiation through *miR-21*. Furthermore, in an inflammatory environment, the expression of *miR-21* was significantly increased when RAW 264.7 cells were exposed to milk-derived sEVs (Figure 6G). The addition of milk-derived sEVs promoted the expression of M2 polarizing factors CD206 and Arg1 while inhibiting the expression of M1 polarizing factor CD86 and inflammatory factor IL-1β in RAW 264.7 cells. However, when *miR-21* was inhibited, contradictory results were obtained (Figure 6). This suggested that milk-derived sEVs promoted RAW 264.7 cell polarization towards the M2 phenotype and inhibited the expression of inflammatory factors through *miR-21*.

## 3. Discussion

CAP is a disease characterized by persistent infection and the resorption of periapical alveolar bone [29]. It has been shown that LPS enhances the activity of osteoclasts and exacerbates the inflammatory response in an experimental mouse model of CAP by promoting the expression of the low-density lipoprotein receptor in macrophages [30]. The control of infection and the restoration of periapical bone defects are essential aspects of CAP treatment [31]. However, due to the complex microbial environment and unique anatomical structures in root canals, inflammation and bone defects may persist [32]. Therefore, further investigation into potential treatment approaches for CAP that promote osteogenesis and possess anti-inflammatory properties is urgently required.

Numerous studies have demonstrated that *miR-21* plays a crucial role in anti-inflammatory responses. For instance, overexpression of *miR-21-5p* in LPS-induced dental pulp cells suppressed pro-inflammatory cytokines by inhibiting *TRAF6* and *PDCD4* and downregulating the *TLR/NF-κB* signaling pathway. This suggested that *miR-21* might inhibit inflammation and contribute to pulpitis [33]. The transfection of *miR-21* mimics inhibited the production of pro-inflammatory factors by activating *NF-κB* in macrophages. Knockout of *miR-21* resulted in enhanced gingival inflammation and alveolar bone loss, indicating that *miR-21* served as a potential target for periodontitis treatment [14]. However, the role of *miR-21* in CAP remains unexplored. In this study, we revealed the downregulation of *miR-21*, as well as of the osteogenic markers OPN and ALP, in human CAP. Conversely, expression of the inflammatory factors TNF-α and IL-1β increased. These findings suggest a potential association between *miR-21* and CAP.

*MiR-21* also plays a crucial role in promoting bone formation [34]. Previous studies have demonstrated that transfection of *miR-21-5p* mimics into MC3T3-E1 cells enhanced the expression of Runx2 and Osterix through the *miR-21-5p/Dec1* axis [35]. Conversely, inhibiting *miR-21-5p* resulted in a decrease in ALP activity, the suppression of mineralization, and the downregulation of BMP2, Runx2, and OCN at both mRNA and protein levels [7]. These findings exhibited similarity to our research. In our study, we also found that *miR-21* was involved in osteogenic differentiation and might influence the expression of OPN and ALP. However, the effect of *miR-21* on the osteogenic differentiation of MC3T3-E1 cells under LPS stimulation remained unclear. Therefore, we further stimulated MC3T3-E1 cells with LPS to induce chronic inflammation and then investigated the impact of *miR-21* on cell proliferation and osteogenic differentiation. Subsequently, we observed that *miR-21* promoted the proliferation of MC3T3-E1 cells and increased the expression of osteogenic genes and proteins. Conversely, inhibition of *miR-21* had the opposite effect. These findings suggest that *miR-21* mimics reversed the inhibitory effect of inflammation on MC3T3-E1 cells and promoted their proliferation and osteogenic differentiation.

Macrophages also play a significant role in the pathogenesis of CAP [36] and are closely associated with inflammation [37]. Macrophages can exhibit two main phenotypes: the M1 phenotype, which promotes inflammation [38], and the M2 phenotype, which possesses anti-inflammatory abilities and contributes to immune regulation and repair processes [39]. The expression of M1 polarizing factors is increased in symptomatic CAP, and the M1/M2 ratio influences the severity of CAP [40]. Previous studies have demonstrated that delivery of *miR-21-5p* inhibited the expression of inflammatory markers in LPS-stimulated RAW 264.7 cells [41]. These findings exhibited similarity to our research. Conversely, controversial research has revealed that overexpressing *miR-21* increased pro-inflammatory cytokines significantly, promoting a pro-inflammatory phenotype, partially involving *PI3K* and *NF-ĸB* signaling pathways [42]. Moreover, the role of *miR-21* in macrophage polarization remains unclear. Therefore, we established an LPS-mediated inflammatory environment to investigate the role of *miR-21* in the polarization of RAW 264.7 cells. We found that under LPS stimulation, *miR-21* might exert an anti-inflammatory effect. To explore whether the anti-inflammatory effect of *miR-21* was related to macrophage polarization, we carried out qRT-PCR, which showed that under LPS stimulation, the expression of M1 polarizing factors increased while the expression of M2 polarizing factors decreased, indicating a polarization of RAW 264.7 cells towards the M1 phenotype. However, transfection of *miR-21* mimics in the inflammatory environment produced contradictory results, indicating that *miR-21* exerted an anti-inflammatory effect and was associated with the polarization of RAW 264.7 cells towards the M2 phenotype. These results indicated that *miR-21* might be a potential target for CAP treatment by promoting osteogenesis and inhibiting inflammation. Nevertheless, despite our research revealing that promoting macrophage M2 polarization suppressed inflammation, scholars have found that laryngopharyngeal reflux (LPR) promoted macrophage M2 polarization, leading to burning mouth syndrome (BMS) [43] and laryngeal squamous cell carcinoma (LSCC) [44]. Therefore, further investigation is needed to explore the detrimental effects of *miR-21* in promoting macrophage M2 polarization.

Our previous studies showed that milk-derived sEVs enhanced the proliferation and differentiation of osteoblasts in vitro and promoted bone repair in a rat skull defect model in vivo. This highlighted their potential for application in bone defect repair to treat CAP [45]. Additionally, milk-derived sEVs effectively inhibited the infiltration of inflammatory cells and attenuated the inflammatory response by inhibiting the *TLR4-NF-κB* signaling pathway and NLRP3 inflammasome activation [46]. Research has shown that sEVs inhibit osteoclast differentiation and alleviate osteoporosis by acting through OPN and *miR-21-5p* [47]. Furthermore, other studies have indicated that milk-derived sEVs are rich in *miR-21* [28]. However, whether milk-derived sEVs promote osteogenesis and inhibit inflammation in CAP through *miR-21* remains unexplored. In our study, we observed that sEVs significantly increased the expression of *miR-21* in MC3T3-E1 cells and RAW 264.7 cells within an inflammatory environment. Treatment with sEVs promoted the expression of osteogenic differentiation marker proteins, but this effect was blocked by the addition of *miR-21* inhibitors. In an inflammatory environment, sEVs promoted the anti-inflammatory M2 polarization of RAW 264.7 cells and inhibited the expression of inflammatory cytokines, effects which were also attenuated by the addition of *miR-21* inhibitors. Therefore, we speculated that milk-derived sEVs would promote osteogenic differentiation of MC3T3-E1 cells, induce M2 polarization of RAW 264.7 cells, and inhibit inflammation through the action of *miR-21*.

However, this experiment has several limitations. First, the specific mechanism by which milk-derived sEVs promote the expression of *miR-21* in cells remains unclear, and whether it involves upstream *LncRNA* is unknown. Second, the downstream targets and signaling pathways of *miR-21* require further exploration. Lastly, there is a lack of animal experiments regarding CAP. Therefore, further exploration of the downstream targets and signaling pathways of *miR-21* should be detected and validated through database analysis and dual-luciferase reporter gene assays. Moreover, animal experiments need supplementation, such as the utilization of a *miR-21* knockout mouse model for CAP. Looking ahead, we intend to utilize engineered milk-derived sEVs loaded with *miR-21* to explore their potential application in CAP.

## 4. Materials and Methods

### 4.1. Sample Collection

This study received approval from the ethics committee of the Affiliated Stomatological Hospital of Dalian Medical University (2022002). Between April 2022 and April 2023, periapical tissue samples were collected from patients diagnosed with CAP. The CAP group consisted of 25 patients who required apical surgery or tooth extraction, while the control (CON) group comprised 25 healthy premolars or third molars extracted for orthodontic reasons or because they were impacted. Among them, 15 cases from the CAP group and 15 cases from the CON group were subjected to qRT-PCR, 6 for Western blot analysis, and 4 for HE staining. The age range of the participants was 24 to 55 years, with a total of 23 females and 27 males. There were no significant differences in sex or age between the two groups (*p* > 0.05). Participants with combined periodontal or periodontal pulp lesions, a history of root canal therapy, systemic diseases, and those using non-steroidal anti-inflammatory drugs or antibiotics were excluded from this study [48].

### 4.2. Hematoxylin and Eosin (HE) Staining

The periapical tissues were fixed in 4% paraformaldehyde and subsequently washed in phosphate-buffered saline (PBS). Dehydration of the tissues was performed using graded alcohols. Xylene was used as a clearing agent to prepare the tissue for further processing. Following the clearing step, the periapical tissues were infiltrated with paraffin wax. Once the infiltration was complete, the tissues were embedded in paraffin wax, and 5 μm continuous sections were obtained using a microtome. HE staining was employed to assess the histological changes in the periapical tissues.

### 4.3. Cell Culture and Induction

MC3T3-E1 and RAW 264.7 cell lines were obtained from the Center for Excellence in Molecular Cell Science (Shanghai, China) and were cultured in α-MEM and DMEM, respectively, supplemented with 10% fetal bovine serum (FBS) and 1% penicillin/streptomycin. Both cell lines were cultured in the T25 cell culture flask and placed at 37 °C in a humidified incubator with 5% CO_2_. For osteogenic differentiation, MC3T3-E1 cells were seeded in six-well plates at 2 × 10^8^ cells/well. Osteogenic induction was performed by adding dexamethasone (10 nmol/L), β-glycerophosphate sodium (10 mmol/L), and ascorbic acid (50 μg/mL) to each well of MC3T3-E1 cells every other day following a 24 h culture period. The induction of LPS involved adding LPS from the *Porphyromonas gingivalis* to the cell culture medium in the range of 0 to 10,000 ng/mL each day.

### 4.4. Cell Transfection Assay

The miRNA mimics and inhibitors were transfected into MC3T3-E1 cells and RAW 264.7 cells with the Lipofectamine 3000 transfection reagent (Thermo Fisher Scientific, Waltham, MA, USA) according to the manufacturer’s instructions. MiRNA mimics and NC were transfected at a concentration of 50 nM, while miRNA inhibitors were used at 100 nM. The cells were incubated for 24 h. Afterward, the transfection medium was replaced with normal growth medium. Total RNA was then extracted after 24–48 h, and protein extraction was performed after 48–72 h.

### 4.5. Cell Proliferation Assay

After transfection of MC3T3-E1 cells or RAW 264.7 cells in an inflammatory environment for 24 h, the cells were resuspended and seeded into 96-well plates at a concentration of 4 × 10^3^ cells per well. Following incubation for 0, 24, or 48 h, the Cell Counting Kit-8 (CCK-8) reagent (APExBIO, Houston, TX, USA) was added to each well at a volume of 10 μL to measure cell proliferation. After incubating for an additional two hours, the optical density (OD) was measured at 450 nm using a spectrophotometer.

### 4.6. Isolation and Characterization of sEVs Derived from Milk

Milk-derived sEVs were prepared according to a previously published protocol [45]. Briefly, milk was collected from healthy cows (Sanhuan Ranch, Dalian, China). Following the defatting process, the skimmed milk underwent centrifugation at varying speeds: first at 13,000× *g* for 30 min, then at 100,000× *g* for 120 min. The resultant middle whey fraction was collected and subjected to further centrifugation at 130,000× *g* for 90 min, followed by 100,000× *g* for 120 min. After filtration, the suspension containing milk-sEVs was obtained through centrifugation at 100,000× *g* for 60 min. The concentration of sEVs was detected using the BCA method. Based on our previous research, 20 µg/mL milk-sEVs were incubated with MC3T3-E1 and RAW 264.7 cells. The milk-sEVs were administered every 24 h. After 24 h, RNA was extracted to assess the levels of osteogenic gene expression and inflammatory cytokines. Following 48 h, the protein expression was tested. The morphology of the sEVs was observed using a transmission electron microscope, the size of the sEVs was measured using nano-flow cytometry, and surface marker proteins were detected by Western blotting using the antibodies anti-CD81 (1:1000, Abcam, Cambridge, MA, USA), anti-CD40 (1:1000, Bioss Antibodies, Woburn, MA, USA), anti-Alix (1:1000, Abbexa Ltd., Cambridge, UK), and anti-TSG-101 (1:1000, Abcam).

### 4.7. Quantitative Reverse Transcription–Polymerase Chain Reaction (qRT-PCR)

Total RNA was extracted using the UNlQ-10 Column Trizol Total RNA Isolation Kit (Sangon Biotech, Shanghai, China). Subsequently, the RNA was converted into complementary DNA (cDNA) using the Evo M-MLV Reverse Transcription Kit (Accurate Biology, Hunan, China). For miRNA reverse transcription, the stem-loop method was employed. The primers used in the reverse transcription process can be found in Table 1. qRT-PCR analysis was performed using SYBR Green Pro Taq and conducted on a Step Two qRT-PCR System (Bio-Rad, Hercules, CA, USA). The obtained data were analyzed using the 2^−ΔΔCt^ method.

### 4.8. Immunofluorescence (IF)

After transfecting MC3T3-E1 cells for 48 h, immunofluorescence staining was performed to visualize specific proteins. Cells were fixed in 4% paraformaldehyde for 20 min and blocked with goat serum at room temperature for 1 h. Cells were then incubated with primary antibodies, including anti-OPN (1:200, Proteintech, Wuhan, China), anti-ALP (1:200, Abcam), and anti-Runx2 (1:200, Abcam) overnight at 4 °C followed by secondary antibody for 1 h at room temperature. A fluorescence microscope (Olympus, Tokyo, Japan) was used to visualize the results.

### 4.9. Western Blotting

Tissue and cell samples were lysed using RIPA buffer to extract proteins, and the protein concentration was determined using a BCA protein assay kit (Beyotime Biotechnology, Shanghai, China). Subsequently, the samples were separated by sodium dodecyl sulfate–polyacrylamide gel electrophoresis (SDS-PAGE) for 1.5 h and then transferred to nitrocellulose (NC) membranes by blotting for 1.5 h. To prevent nonspecific binding, the NC membranes were blocked with fat-free milk at room temperature for 2.5 h and then incubated overnight at 4 °C with the primary antibodies anti-OPN (1:1000, Proteintech, Rosemont, IL, USA), anti-ALP (1:1000, Abcam), and anti-Runx2 (1:500, Abcam). Finally, the membranes were incubated with the secondary antibody (1:5000, ABclonal, Woburn, MA, USA) for 1 h. A gel imaging system (Bio-Rad, Hercules, CA, USA) was used to visualize the protein bands.

### 4.10. Statistical Analysis

Data are expressed as mean ± SEM. SPSS Statistics (version 17.0) software was used for all statistical analyses. The experiments were repeated independently a minimum of three times. GraphPad Prism 7.0 was employed to create the statistical graphs. Student’s *t*-test or one-way ANOVA was performed to verify statistical significance. A *p*-value < 0.05 was statistically significant.

## 5. Conclusions

In summary, our results show that *miR-21* increased the expression of osteogenesis-related genes and M2 polarization genes while decreasing the expression of M1 polarization genes and inflammatory cytokines. This indicated that *miR-21* might be a potential target for CAP treatment. In the regulation of osteogenic differentiation and anti-inflammatory effects by milk-derived sEVs, *miR-21* played a crucial role since the suppression of *miR-21* markedly reduced the regulatory effects of sEVs. Our discovery indicates for the first time that milk-derived sEVs might represent a novel therapeutic tool for CAP by targeting *miR-21*. For instance, the development of targeted drugs for *miR-21*, the design of delivery systems for sEVs intended for root canal disinfection and filling, or the utilization of a scaffold-loaded approach to directly implant them into the bone defect associated with CAP. Further studies are required to elucidate the specific mechanisms, downstream targets, and signaling pathways and to confirm the findings in vivo by animal experiments.

## Figures and Tables

**Table 1:** List of primers.

Primers	Base Sequence
*U6 F*	5′-GTGAAGGTCGGAGTCAACG-3′
*U6 R*	5′-TGAGGTCAATGAAGGGGTC-3′
*miR-21-5p-F*	5′-TCGCCCGTAGCTTATCAGACT-3′
*miR-21-5p-R*	5′-CCAGTGCAGGGTCCGAGGT-3′
*m-OPN-F*	5′-GCTCAGCACCTGAATGTACC-3′
*m-OPN-R*	5′-CCTCGGCTCGATGGCTAGC-3′
*m-ALP-F*	5′-TGAATCGGAACAACCTGACTGA-3′
*m-ALP-R*	5′-GAGCCTGCTTGGCCTTACC-3′
*m-IL-1β-F*	5′-CCAGGATGAGGACATGAGCAC-3′
*m-IL-1β-R*	5′-TGTTGTTCATCTCGGAGCCTGTA-3′
*m-TNF-α-F*	5′-GGTGCCTATGTCTCAGCCTCT-3′
*m-TNF-α-R*	5′-ACGTGGGCTACAGGCTTGTC-3′
*m-CD86-F*	5′-TTTCCTCCAAACCTCTCAATTTCA-3′
*m-CD86-R*	5′-TGGGCCTGCTAGGCTGATT-3′
*m-CD206-F*	5′-AGCTTCATCTTCGGGCCTTT-3′
*m-CD206-R*	5′-CCCTTGGGTTGAGGATCCAT-3′
*m-Arg1-F*	5′-TGGGTGACTCCCTGCATATCT-3′
*m-Arg1-R*	5′-CACCTTGGTCTTGGAGCTTATTAAA-3′

## Data Availability

All data used in this study are included in this article.
